# The impact of anti-tobacco legislation on birth weight in Peru

**DOI:** 10.1186/s41256-020-00136-5

**Published:** 2020-02-28

**Authors:** Patricia Mallma, Cesar Carcamo, Jay S. Kaufman

**Affiliations:** 1 Epidemiology, HIV and STD Unit, School of Public Health and Administration, Universidad Peruana Cayetano Heredia, Lima, Peru; 2 Department of Epidemiology, Biostatistics, and Occupational Health, McGill University, Montreal, Canada

**Keywords:** Tobacco control, Peru, Low birth weight, Prematurity and small gestational age

## Abstract

**Background:**

Tobacco exposure remains a significant issue for public health, especially for pregnant women. It increases the risk for premature labor, low birth weight and small for gestational age (SGA), among other effects. To reduce these risks, many countries have enacted public policies to curb tobacco exposure. Peru enacted anti-tobacco laws that forbid smoking in public places, require prevention text and images in products and publicity, along with restriction of sales to adults. We evaluated the effect of the implementation of this law on newborn outcomes: birth weight, prematurity and SGA.

**Methods:**

This was a quasi-experimental study that utilized data from the Peruvian Live Birth Registry. Children born to mothers from urban areas were the intervention group, while children born to mothers from rural areas were considered the control group. Only singletons with information on birth weight and gestational age, born to mothers aged 12 to 49 years were included in the study. In addition, newborns with birth weights greater than + 4 standard deviations (SD) or less than − 4 SD from the gestational age-specific mean were excluded. To measure the effect of legislation on birth weight we performed a difference in differences analysis.

**Results:**

A total of 2,029,975 births were included in the analysis. After adjusting for characteristics of the mother and the child, and contextual variables, the anti-tobacco law in Peru reduced the incidence of prematurity by 30 cases per 10,000 live births (95% CI: 19 to 42).

**Conclusions:**

The reform had negligible effects on overall birth weights and on the incidence of SGA. This modest result suggests the need for a more aggressive fight against tobacco, prohibiting all types of advertising and promotion of tobacco products, among others measures.

## Introduction

During the last century, heavy advertising was associated with a significant increase in tobacco consumption and exposure to tobacco smoke. By the end of the twentieth century, smoking was considered a worldwide pandemic [1].

Tobacco is well known as a risk factor in the development of many diseases [1–8], and premature death [9–11]. Its negative effects have been demonstrated both, in animal experiments and in humans [1, 2].

Tobacco exposure is associated with preterm birth, small for gestational age (SGA) and low birth weight, which can result in chronic disease and infant death [12–18]. Being born premature is the second leading cause of death in the first 5 years of life and is the main cause of death within the first month of life [19]. Compared to a normal-weight newborn, low birth weight newborns will have a greater probability of dying in the first month of life, as well as being more predisposed to disease [20].

In order to reduce these risks, many countries have created public policies to protect the population from exposure to tobacco, including banning smoking in public places, the inclusion of images and warning phrases about the health effects of tobacco consumption on cigarette boxes, increasing cigarette taxes, etc. [21]. Many studies have demonstrated the impact of these legislations among adults, finding that anti-tobacco laws improved air quality, reduced tobacco biomarkers, and improved the respiratory health of bar workers in Korea, Ireland, United States and Spain [22–25], as well as improved air quality and respiratory health for restaurant workers from Portugal [26].

Anti-tobacco laws have shown positive effect on hospitalizations for respiratory tract infection and asthma in children [27–30]. Likewise, anti-tobacco laws have reduced adverse effects on newborns from Canada, United States, Ireland, England, Belgium, Norway, Spain, the Netherlands, Switzerland, Hungary, Scotland and Uruguay [29–46]. Nevertheless, the information from developing and middle-income countries is still limited.

In Latin American countries, laws have also been created to reduce tobacco exposure. In Peru, a series of restrictions was passed into law after 1991 [47–52]. As a consequence of Peru signing the Framework Convention on Tobacco Control, legislation was enacted in 2006 prohibiting smoking in public places. In 2010, a new anti-tobacco law was enacted which modified certain aspects of the 2006 law, making the provisions stronger: smoking areas were removed, warning phrases and images on both sides of tobacco products were required, and sales of packages with less than 10 units were forbidden.

Like most Latin American countries, the anti-tobacco laws in Peru have not been formally evaluated with respect to an objective health outcome. We are therefore interested in evaluating the effect of this policy on birth weights, prematurity and SGA.

## Methods

The study design was quasi-experimental, due to the non-random nature of the intervention. We utilized data from the Peruvian Live Birth Registry, from 2005 to 2016. Children of mothers residing in urban areas were considered the intervention group, since bars and restaurants in urban areas would be affected by the new smoke-free regulations. These exposed children were compared to children of mothers residing in rural settings, where there would be few public places affected by the new prohibitions and little enforcement of the new law. Between 2016 and 2018, district and national level authorities in Peru carried out at least 175 unannounced visits to restaurants with the objective of enforcing anti-tobacco laws. None of these enforcements were carried out in rural areas [53–55]. Likewise between 2009 and 2019, of 49 municipal ordinances for anti-tobacco law enforcement ruled by the same number of municipalities in that period, all but one occurred in urban districts [56, 57]. Besides this single district, we have not identified any other effort to enforce anti-tobacco laws in rural areas. It was assumed therefore that the intervention started in urban areas when the anti-tobacco law was enacted, so that all urban pregnancies since the legislation date were exposed to the new legal regime. Smoking trends in Peru are shown in supplementary support S1.

### Study population

Registration of all births is mandatory in Peru [58]. Registration is carried out by the health professional (usually a professional midwife) who assisted the delivery. Births can be registered immediately after birth, and there is no maximum age. Since 2012 data can be entered directly into an electronic form in places where Internet access is available. For year 2015 the coverage of the registry is estimated to be 72% [59]. The registry is maintained on Ministry of Health’s servers. We included all live births registered as occurring between 2005 and 2016. This registry is managed by the “National Institute of Statistics and Informatics” (INEI).

We considered the following inclusion criteria: single births, with complete information on birth weight and gestational age, as well as information on the co-variables of interest, and born from mothers between 12 and 49 years of age. We also excluded birth weights deviating more than 4 standard deviations from the corresponding mean for each recorded gestational age [60], using as a reference the population of Canada [61].

### Public interventions to reduce exposure to tobacco

In 2003, the World Health Organization (WHO) developed the “Framework Convention on Tobacco Control” (FCTC) to “protect present and future generations from the devastating health, social, environmental and economic consequences of smoking and exposure to smoke.” So far, 181 countries have adhered to this Convention, committing themselves to generate national strategies, plans and policies to reduce exposure to tobacco [62].

Peru also signed the CMCT in 2004. Prior to this agreement, there were already some laws to reduce exposure to tobacco in this country [47–50], but it was not until 2006 that law No. 28705, the “General Law for the Prevention and Control of Risks of Tobacco Use” was enacted. This law consists of four chapters that cover: 1) prevention and protection of the population, through regulations that forbid smoking in closed public and private buildings (bars, restaurants, movie theatres, governmental buildings, etc.), as well as in public transport vehicles, where people could be exposed to second hand smoke. In addition, it required hotels, restaurants and other public places to maintain a specific smoking area. This chapter also included the obligation of enclosed spaces to use signs with the following inscription: “Smoking in public places like this is prohibited according to Law No 28705”, “Smoking is harmful to health, smoke also harms non-smokers”; 2) packaging and labeling of tobacco products: the law stipulates that 50% of one of the faces of the packages of these products must be printed with phrases or pictures describing health harm caused by tobacco consumption, and forbids the inclusion of words like “light”, “ultra-light”, “soft”, and “super soft; 3) commercialization: the law prohibits the sale of tobacco products in health or educational establishments and requires every place that sells tobacco products to have a sign with the following inscription: “Smoking is harmful to health *–* Sale forbidden to people under 18 years old”; finally, in chapter 4) advertising, promotion, and sponsorship of tobacco, the law determines that all advertising of tobacco products must include warning phrases about the health effects of smoking, and that tobacco brands are prohibited from sponsoring events aimed at minors. The law also included penalties if the established regulations were not obeyed [51]. Finally, to reconcile the FCTC with the law No. 28705, some aspects were modified in 2010 and law No. 29517 was created. This second piece of legislation ended the option of having special smoking areas in hotels, bars, restaurants, etc., as well as requiring these places to have the following inscription: “Smoking in public places is prohibited because it is harmful to health”, “100% smoke-free environment”. Additionally this law required 50% of both faces of the packages of tobacco products to be printed with warning phrases and pictures describing the harmful effects of tobacco consumption, and prohibited the sale of packages containing less than 10 units of tobacco products [52].

### Implementation of the anti-tobacco law

In Peru, the implementation of new laws requires the approval of regulations, technical standards, which are detailed in supplementary support S2. The regulation considers measures related to each chapter of Law No. 28705. For Chapter 1, it specifies that Municipalities and the Ministry of Health will be responsible for conducting inspections on tobacco control in workplaces, restaurants, bars, hotels, etc., and for levying fines on establishments that do not obey the law. For the chapter on packaging of tobacco products and advertising of tobacco products, the regulation lists health warning messages that products and advertising signs must contain, as well as their size. For the commercialization chapter, the regulation specifies the size of the warning signs to be posted in places where tobacco products are sold. The regulation also establishes sanctions for non-compliers [63]. Although the law 28,705 was approved in 2006, its regulation was not approved until July 5, 2008. Therefore the date of the impact evaluation for our study was assigned as the date of approval of this regulation. A secondary evaluation uses the date of publication of the Law No. 29517 (April 2, 2010) as a sensitivity analysis, along with plausible lags in enforcement. The implementation of the pictures and warning messages about health effects of tobacco consumption on the packaging, advertising of cigarettes and other products made with tobacco occurred in 2009.

### Exposure variables

**2008 law**: Coded as “1” for births occurring after July 5th, 2008 (date of publication of the regulation for the law No. 28705) from mothers residing in urban areas and “0” otherwise.

### Outcomes variables

We evaluated three outcome variables using data from the Peruvian Live Birth Registry: (i) **Birth weight** in grams, (ii) **Preterm birth,** coded as “1” for gestational age less than 37 weeks and “0” otherwise, and **(iii) Small for gestational age,** coded as “1” for birth weight lower than the corresponding 10th percentile for gestational age and “0” otherwise, using as the reference the data on Canadian births [61, 64].

The Registry did not contain information about the method of ascertaining gestational age (date of last period, ultrasound, Capurro method, etc.). We consulted health personnel in charge of the database, and found that clinical estimates were based on information available, whether reported last menstrual period, ultrasound or Capurro Method at the discretion of the attending physician.

### Covariates

Other factors included in the analysis were: maternal factors (age, marital status, level of education, parity), child factors (gender, year of birth, place of delivery, person that assisted the delivery), district level covariates (urban setting versus rural, poverty in quintiles, and altitude). The 2011 official classification was used to define the urban/rural status of the district. Districts were classified as rural when their municipalities were not located within the district, or when more than 50% of their population live in rural areas [65]. The percentage of poverty of the district of residence of the mother [66] was assigned to each newborn, and this value was then categorized in quintiles ranging from 1 for those living in the richest districts to 5 for the poorest. The altitude was measured at the main plaza at the capital of the district [67].

### Statistical analysis

Data were analyzed using STATA 15.1 (StataCorp, College Station, Texas). Records with missing data were for gestational age, birth weight, place of birth, parity, education of mother, and gender of newborn were excluded from the analysis. We first explored urban/rural differences in the outcomes and co-variables of interest. We assessed parallel pre-intervention trends in the treated and control group prior to carrying out analyses for the three outcomes (see supplementary support S3).

The outcome for the first model was birth weight in grams as a continuous variable. Multivariate analysis was performed using difference in differences in a linear mixed effects model. District characteristics were included at the cluster level. Due to the lack of a linear relationship between birth weight and mother’s age, a quadratic term for maternal age was included in the model. Likewise, the shape of the relationship between birth weight and altitude of the district of residence was verified as a linear effect (see supplementary support S4). We analyzed the number of pregnancies of the mother as a categorical variable. Thus the equation for the linear mixed effects difference in differences model is as follows:

1 $$ {y}_{id}={\beta}_0+{\beta}_1\bullet antitobacco la{w}_{id}+{\beta}_2{urban}_{\mathrm{d}}+{\beta}_3\bullet antitobacco{law}_{id}\bullet {urban}_d+{\beta}_4\bullet i. year\_{birth}_{id}+\delta \bullet {Covariates}_d+\alpha \bullet {Covariates}_{id}+{\varepsilon}_{id}+{\mu}_d $$

Where:

- *i* = child ID
- *d* = district’s ID
- *y*_*id*_ = birth weight of child *i* born to district *d*
- *antitobaccolaw*_*id*_ *=* coded as 1 for children born after the implementation date of the law and 0 otherwise.
- urban_*d*_= coded as 1 for households located in urban areas or 0 otherwise
- *Covariates*_*d*_ = district characteristics: poverty, altitude (in meters above sea level).
- *Covariates*_*id*_= maternal characteristics: age, marital status, level of education, parity; and child characteristics: gender, year of birth, place of delivery, person that assisted the delivery.
- *ε*_*id*_= non-observed characteristics of the district.
- *μ*_*d*_ = non-observed characteristics of the child.
- *β*_3_ = effect of the anti-tobacco law on birth weight. A positive sign of the coefficient would correspond to a gain in birth weight.

The outcomes for the second and third models were prematurity (< 37 weeks of gestational age at birth) and SGA. They were evaluated using mixed effects logistic regression. The average marginal effects were estimated in order to obtain the effect of the anti-tobacco law on the absolute scale. The mixed effects logistic differences in differences model used is:

2 $$ logit\left[\left(\frac{p}{1-p}\right)\right]={\beta}_0+{\beta}_1\bullet antitobacco{law}_{id}+{\beta}_2\bullet {urban}_d++\delta \bullet {covariates}_d+\alpha \bullet {covariates}_{id}+{\varepsilon}_{id}+{\mu}_d $$

Where:

- *p* = probability of prematurity or SGA.

The remaining variables correspond to those presented in eq. 1, except altitude. Altitude was analyzed as a linear and quadratic term for the prematurity model and as a linear term for SGA (see supplementary support S4). For the three outcomes (birth weight, prematurity and SGA) only information from the 3 years before and 3 years after the date of the 2008 regulation was included. We expect that this would reduce the risk of contamination from the effect of other policies implemented in neighboring years. As a sensitivity analysis, three additional models were fit to explore the effect of the 2010 law. In addition all multivariate models were adjusted for potential confounders.

## Results

### Birth weight trends according to area of residence

After applying our inclusion and exclusion criteria to the 4,965,825 births recorded in the database for the 2005–2016 period, we were left with 4,742,253 births for the first part of the analysis.

**Fig. 1:**
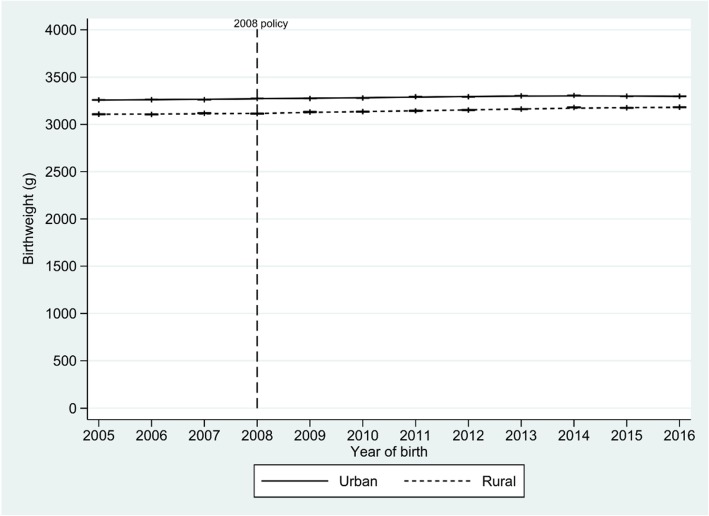
Mean birth weight trends by year and area of residence, Peru, 2005–2016. Dots represent mean birthweight (g) and vertical lines represent 95% confidence intervals for the mean

Figure 1 shows the trend in births weights according the child’s residence area for the period 2005–2016. Visually, in both rural and urban areas there is a subtle upward trend in birth weight from 2005 to 2016 in rural areas, and to 2014 in urban areas.

**Fig. 2:**
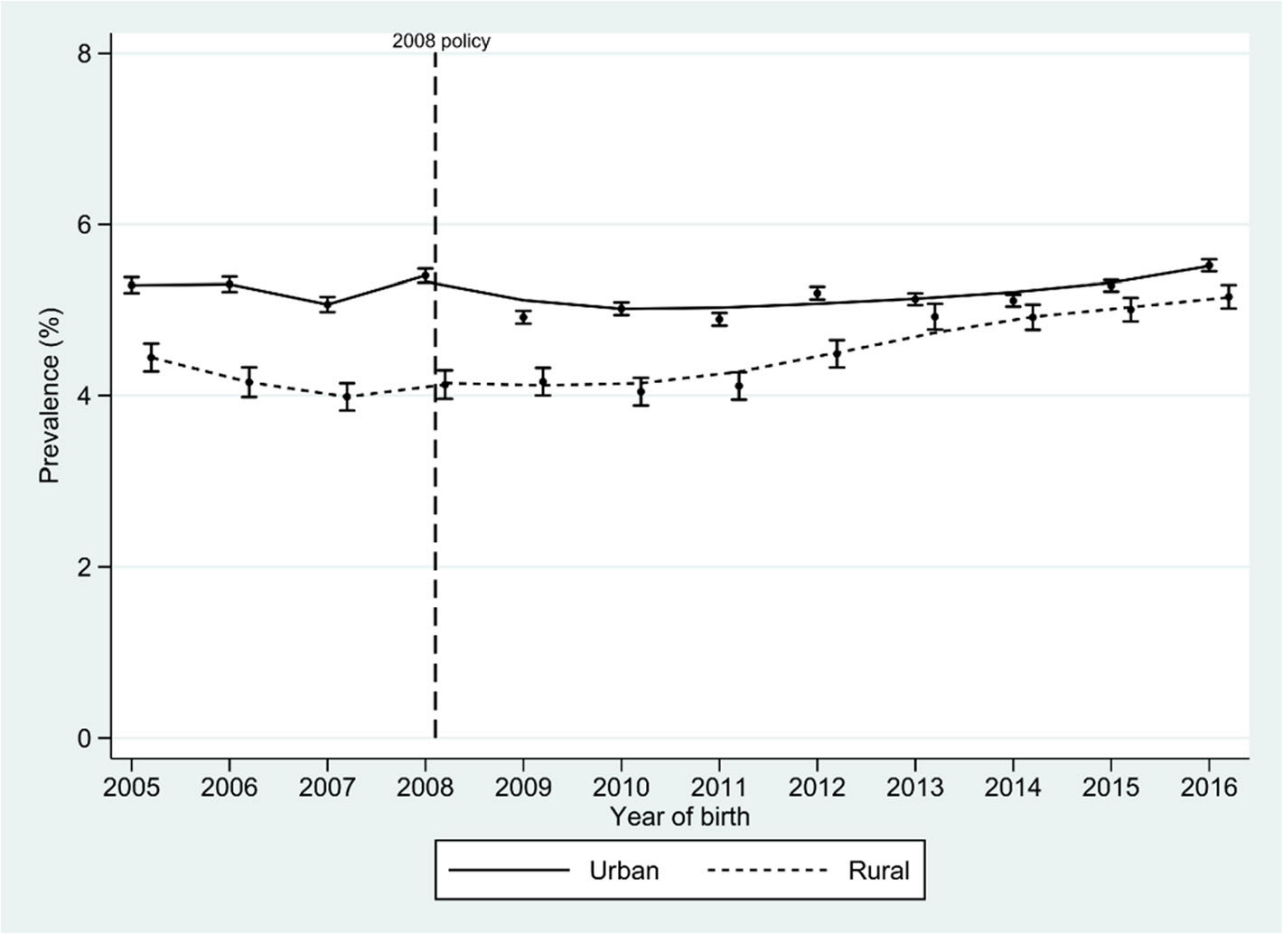
Trends in prematurity prevalence by year and area of residence. Peru, 2005–2016. Dots represent proportion with low birthweight (g) and vertical lines represent 95% confidence intervals for the proportion

**Fig. 3:**
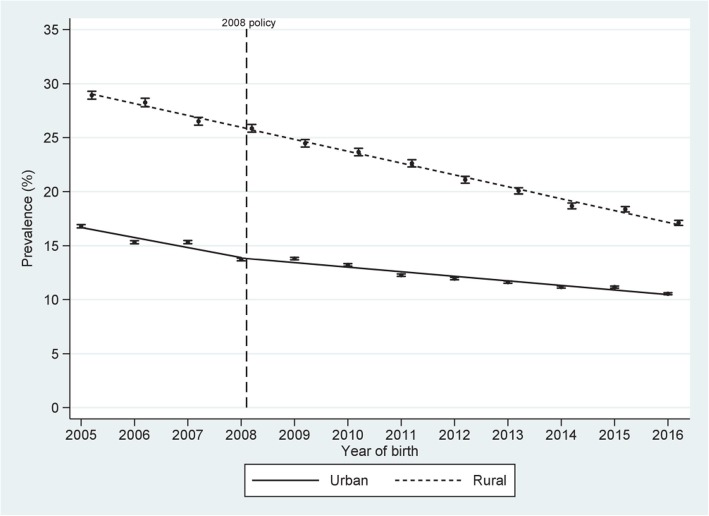
Trends in small for gestational age prevalence by year and area of residence. Peru, 2005–2016. Dots represent proportion with low birthweight (g) and vertical lines represent 95% confidence intervals for the proportion

Figure 2 shows the trend in the percentage of prematurity. There is an apparent stationary trend before to 2010. In rural areas, there is an upward trend after 2010. In the urban area, there was a steady trend after 2010 and after 2014 a trend upwards. A decreasing trend is observed for the percentage of SGA before and after 2010 in both areas (Fig. 3).

The linear regression model found no interaction between time and residence area prior to 2008 and 2010, indicating similar time trends for the two groups in birth weight, prematurity and SGA (see support information S3), as required for the validity of the difference in differences analysis.

### Effect of the implementation of the anti-tobacco law

After excluding births prior to 2005 and after 2013, 2,729,681births remained for the second part of the analysis. The average birth weight and prevalence of prematurity were lower in rural areas compared to urban areas, whereas the percentage of SGA in rural areas was higher than in urban areas. Among characteristics of the child, there were a higher percentage of births assisted by health professionals in urban areas, as well as a higher percentage of institutional deliveries. In terms of maternal characteristics, a higher level of education was observed in urban areas. Poverty levels and altitude were higher in rural areas (Table 1).

**Table 1:** Characteristics of the births in Peru, July 2005 – April 2013

	Urban	Rural
	(*N* = 2,281,689)	(*N* = 447,992)
	n (%)	n (%)
*Outcomes*		
Birth weight, mean (SD)	3278 (485)	3130 (449)
Prematurity	116,994 (5.13)	18,794 (4.20)
Small for gestational age	309,358 (13.56)	110,018 (24.56)
*Characteristics of the child*		
Gender		
Male	1,168,784 (51.22)	228,511 (51.01)
Female	1,112,905 (48.78)	219,481 (48.99)
Gestational age, mean (SD)	38.91 (1.54)	39.00 (1.41)
Person that assisted the delivery		
Health professional	2,184,822 (95.76)	342,851 (76.53)
Health technician	15,946 (0.70)	30,390 (6.78)
Midwife	42,538 (1.86)	33,456 (7.47)
Another person	38,383 (1.68)	41,295 (9.22)
Place of birth		
Institutional	2,145,301 (94.02)	333,841 (74.52)
Year of birth		
2005	104,161 (4.57)	26,791 (5.98)
2006	240,618 (10.55)	50,312 (11.23)
2007	233,966 (10.25)	57,719 (12.88)
2008	288,645 (12.65)	54,909 (12.26)
2009	321,822 (14.10)	59,082 (13.19)
2010	332,504 (14.58)	57,214 (12.77)
2011	325,377 (14.26)	59,221 (13.22)
2012	335,468 (14.70)	62,584 (13.97)
2013	99,128 (4.34)	20,160 (4.50)
*Characteristics of the mother*		
Maternal age, mean (SD)	27.09 (6.65)	26.51 (7.19)
Level of education		
No education	44,458 (1.95)	51,677 (11.54)
Primary	363,717 (15.94)	209,856 (46.84)
Secondary	1,178,961 (51.67)	160,834 (35.90)
Superior non-university	392,305 (17.19)	19,029 (4.25)
Higher university	302,248 (13.25)	6596 (1.47)
Marital status		
Cohabitation	1,467,183 (64.31)	308,428 (68.85)
Married	567,405 (24.87)	105,521 (23.55)
Previously joined	10,607 (0.46)	3224 (0.72)
Single	236,494 (10.36)	30,819 (6.88)
Number of pregnancies, mean (SD)	2.33 (1.53)	3.07 (2.23)
*Characteristics of the residence district of the mother*		
Poverty quintile^a^		
Richest (0.00 to 9.78%)	599,890 (26.29)	8615 (1.92)
Richer (9.79 to 16.92%)	516,832 (22.65)	9820 (2.19)
Middle (16.93 to 22.80%)	540,268 (23.68)	17,097 (3.82)
Poorer (22.81 to 40.99%)	435,716 (19.10)	81,233 (18.13)
Poorest (41.00 to 100.00%)	188,983 (8.28)	331,227 (73.94)
Altitude, mean (SD)	900 (1302)	2304 (1345)

*SD* Standard deviation

^a^Numbers in parenthesis represent the lower and upper limit for the percent of the population living in poverty at the districts included in the quintile

To evaluate the effect of the 2008 law we considered 2,029,975 births from 2005 through 2011.

Table 2 shows the effects of the implementation of the law on birth weight, prematurity and SGA, crude and adjusted. The crude estimates showed significant reductions for all 3 outcomes. After adjusting for mother’s age, level of education, marital status, and parity, newborn year of birth, gender, and place of delivery, health care provider for childbirth, area of residence, poverty quintiles, and altitude, the 2008 law resulted in a negligible reduction in birth weight of 3.10 g. (95% CI: − 6.57, 0.37), a negligible reduction in SGA (a decrease of 6 cases per 10,000 live births, 95% CI: − 25, + 13), and a significant reduction in prematurity (30 cases per 10,000 live births, 95% CI: 19, 42).

**Table 2:** Estimated effect adjusted of anti-smoking legislation on birth outcomes in Peru

Outcome	2008 legislation	
	Crude difference (95% CI)	Adjusted difference (95% CI)
Birth weight (g)	−4.32 (− 7.83, − 0.81)	− 3.10 (− 6.57, 0.37)
Prematurity (%)	− 0.29 (− 0.35, − 0.23)	−0.30 (− 0.42, − 0.19)
Small for gestational age (%)	−1.81 (− 1.95, − 1.68)	−0.06 (− 0.25, 0.13)

Models adjusted for the following variables: mother’s age, level of education, marital status, and parity, newborn year of birth, gender, place of birth, health care provider for childbirth, area of residence, poverty quintiles, and altitude

### Sensitivity analysis

After adjusting the same covariates used for the 2008 law, the adjusted effect of the 2010 law was a negligible gain of 0.85 g (95% CI: − 2.56, 4.25) in birth weight and a negligible reduction in prevalence of SGA (4 cases per 10,000 live births, 95% CI: − 23, + 14), and again a significant reduction in the proportion of prematurity by 25 cases per 10,000 live births (95% CI: 13, 37).

## Discussion

The data analyzed from the database of live births in Peru, shows that the 2008 and 2010 anti-tobacco laws in Peru do not have a discernible effect on birth weights and proportion of SGA, however, we demonstrated a modest effect of these laws in reducing the proportion of premature births by approximately 30 cases per 10,000 live births.

We identified five studies that reported similar results regarding negligible effects on birth weights after implementing anti-smoking laws in Norway, Ireland, two studies in the United States, and Uruguay [38, 39, 43, 46, 68]. Other studies in USA, England, and Canada showed significantly increased birth weights [40, 42, 45]. A study in Hungary found an important gain in birth weight (55.5 g) in newborns of female workers of restaurants and bars after the implementation of the law [33]; and a study in USA found a 7 g reduction after the implementation of Local Smoking Ordinances [69].

With regards to the premature births proportion, eight studies in Belgium, Scotland, Ireland, Spain, England, Canada, and in two USA states found a positive effect, like our study [34–36, 38, 40–42, 45]. However, seven studies in Netherlands, Switzerland, Hungary, Norway, and 3 in the USA failed to detect an impact [31–33, 39, 46, 68, 70]. Studies in Hungary and Switzerland did not find an effect in preterm births, but did detect a positive effect in very-preterm deliveries [32, 33]. The positive effect observed in our study regarding the risk of prematurity (0.25%) is modest compared to the reduction reported in the studies cited: 23% by Page in the state of Colorado in USA, 25% by Kabir in Ireland, 12% by Mackay in Scotland, 4.5% by Simon in Spain, 4.0% by Bakolis in England, 3.5% by Cox in Belgium, 1.5% by Bartholomew in the state Virginia West in USA, but similar to what McKinnon found in Canada (0.31%).

As in the case of our study, w study showing no effect in the proportion of SGA, in the United States [46], while six in Netherlands, Scotland, Ireland, Spain, England and Canada showed a reduction in SGA following the introduction of anti-tobacco laws [31, 36, 37, 41, 42, 45].

In countries where the studies identified an effect of the anti-tobacco laws on birth weights, preterm delivery or SGA, smoking is generally more common than in Peru. The average number of cigarettes smoked per person per year for these countries is: 2060 for Hungary, 2441 for Belgium, 828 for Scotland, 976 for Ireland, 1017 for the USA, 828 for England, 1460 for the Netherlands, 1021 for Canada, and 1499 for Spain [71]. This findings contrast with the average of 98 cigarettes smoked per person per year in Peru, and could explain the modest effect found in our study. In the same way, while our study examines the effects of only the aspects of the law governing public spaces and labelling, the study in the Netherlands assessed the combined effects of a similar law, plus increased tobacco taxes and a mass media campaign [31]. The positive effect of taxes on tobacco on newborns has been reported elsewhere [46, 68].

The use of rural areas as a control group could be a relative limitation. It was used under the assumption that the law would have little effect in these areas, as there is no much smoking in these areas, and there are fewer closed spaces that could be considered public areas. There is also no active enforcement of these laws outside of major cities in Peru [53, 54, 57]. Another limitation is that the database does not include information on maternal medical conditions (such as diabetes mellitus, hypertension, etc.) known to affect health outcomes of the newborn [72–76]. Additionally, the regulations of the Ministry of health regarding the registration of births in this database do not specify the method to be used for the determination of gestational age, which could alter the results. Finally, Canadian births were used as a reference, but the use of the INTERGROWTH-21st instead [77], lead to no significant changes in the results. The others sensitivity analysis continued to show that adjusted estimates for birth weight remained very small. When child-level covariates are not adjusted, the *p*-value decreases, but nonetheless we think that this value is under-adjusted. With respect to preterm birth when child-level covariates are not adjusted, this estimate becomes 28 prevented cases per 10,000 births, which is a negligible change. With the 6 months lag, this gets even stronger, to 44 prevented cases per 10,000 births. At a 1 year lag, this drop to 13 prevented cases per 10,000 births, but remains statistically significant. For SGA when child-level covariates are not adjusted, this becomes 3 more cases per 10,000 births, not distinct from 0, but with the 6 months lag increases SGA cases significantly. At a 1 year lag, this drops to 23 prevented cases per 10,000 births (see supplementary support S7).

The main strength of our study is the use of national databases, with a high coverage that went from 53.74% in 2005 to 83.40% in 2016 [78], which allow for extrapolations within Peru.

## Conclusions

The implementation of anti-tobacco laws in Peru was associated with a small but significant reduction in the frequency of prematurity. In spite of our modest results, the abundance of data supporting the positive effect of anti-tobacco laws on delivery outcomes means that at least a subpopulation of children born from high risk women likely benefitted from the anti-tobacco laws in Peru.

The evidence found in this study justifies the implementation of anti-tobacco laws for the benefit of public health, with the promotion of 100% smoke-free environments. This should include the fight against smoking, prohibiting all types of advertising, promotion of tobacco products, as well as the sponsorship of all kinds of activities or events, to prevent the initiation of smoking among children and adolescents.

## Supplementary information

**Additional file 1.** Supporting Information.

## Data Availability

The dataset used in this study is not publically available, but is available upon request from the Peruvian Institute of Statistics and Informatics.

## Abbreviations

FCTC: Framework Convention on Tobacco Control
ID: Participant Identifier
INEI: National Institute of Statistics and Informatics
INTERGROWTH-21st: The International Fetal and Newborn Growth Consortium for the twenty-first Century
SD: Standard deviations
SGA: Small gestational for age
WHO: World Health Organization
