# Supplementary material for: The impact of anti-tobacco legislation on birth weight in Peru
Source: Glob Health Res Policy. 2020 Feb 28;5:5. doi: 10.1186/s41256-020-00136-5 (PMC7048150; doi:10.1186/s41256-020-00136-5)
Supplement: Supplementary file 1 — Additional file 1. Supporting Information. [file 41256_2020_136_MOESM1_ESM.zip › Anti-tobacco Peru-Supplementary_GHRP.docx]

SUPPORTING INFORMATION

S1. Trend of smoking in Peru

The Centre for Information and Education for the Prevention of Drug Abuse (CEDRO) has been conducting studies on the use of drugs in the urban population of Peru, tobacco consumption is one of them. The first studies were conducted in the population between the ages of 12 to 50 years, but from 2001, this range was extended to 64 years. It is observed in the prevalence of life (tobacco consumption at least once in the life) a slightly ascending behaviour until 2005, and then a tendency to reduction. Similar behaviours are observed in the prevalence of consumption in the last 12 months (year) and in the last 30 days (current) (figure S1).

S2. Implementation of anti-tobacco public policy

**Table S1. Anti-tobacco public policy in Peru**

| **Publication date** | **Law/Regulation/Technical Standard** | **Summary** |
| --- | --- | --- |
| April 6, 2006 | Law No. 28705: “General Law for the Prevention and Control of Risks of Tobacco Use” | First law enacted after Peru adheres to the FCTC. |
| July 5, 2008 | Supreme Decree No. 015-2008-SA. Regulation of Law N ° 28705, General Law for the Prevention and Control of the Risks of Tobacco Consumption. | Regulation that "regulates the marketing of tobacco products, ensures that its advertising, promotion and marketing is directed only to adults, and establishes the mechanisms of control and administrative sanction."^[[1]](#footnote-1)^ |
| January 14, 2010 | Supreme Decree No. 001-2010-SA. Modification of the Regulations of Law No. 28705. | Mainly stipulates that the smoking area is modified (reduced) in public places. The table of infractions and penalties is presented.^[[2]](#footnote-2)^ |
| April 2, 2010 | Law No. 29517. Law amending Law No. 28705. | Law No. 28705 is modified, so that it is adapted to the FCTC. |
| January 15, 2011 | Supreme Decree No. 001-2011-SA. Modification of the Regulations of Law No. 28705. | The following points were implemented: The last repeals articles of the regulation that allowed for special areas for smokers in public facilities. Activities are determined to perform inspections of 100% smoke-free environments. The table of infractions and penalties is modified.^[[3]](#footnote-3)^ |
| May 28, 2012 | Ministerial Resolution No. 415-2012/MINSA. NTS N ° 094-MINSA/DIGESA. V.01. Technical Health Standard for Inspections of 100% smoke-free environments. | The aim of "Establishing the procedure for carrying out inspections in compliance with the current regulatory framework, for the verification of 100% smoke-free environments".^[[4]](#footnote-4)^ |

Figure S2 shows the chronology of the anti-tobacco public policies.

**Figure S2. Implementation of anti-tobacco public policy in Peru**

S3. Flow diagram of the number of participants who entered the analysis

Figure S3: Flow diagram

**Peruvian Live Birth Registry 2000-2016**

(n=6,770,044)

**Excluded (n=2,027,791**)

- 1,804,219 without gestational age in weeks (all records from 2000 to 2004)
- 223,572 did not meet inclusion criteria

Included in the analysis of trends (n=4,742,253)

**Sensitivity analysis**

**Effect of the anti-tobacco law**

**Urban (n=1,692,153)**

- Births analyzed: 3 years before and 3 years after the date of publication of the regulation of the 2006 anti-tobacco law

**Urban (n=1,876,599)**

- Births analyzed: 3 years before and 3 years after the date of publication of the anti-tobacco law 2010

**Rural (n=355,429)**

- Births analyzed: 3 years before and 3 years after the date of publication of the 2010 anti-tobacco law

**Rural (n=337,822)**

- Births analyzed: 3 years before and 3 years after the date of publication of the regulation of the 2006 anti-tobacco law

S4. Evaluation of parallel trends before enactment of anti-tobacco laws

In preparation for the use of the difference in differences model, the assumption of parallelism in birth weight, prematurity and small for gestational age prior to the intervention was assessed in rural and urban areas. The assessment was made through a multiple linear regression model:

$$y_{i}= \beta_{0}+\beta_{1}time+\beta_{2}urban+\beta_{3}(time\times urban)$$

Where:

i: child

$y_{i}$: outcomes of newborns (birth weight average monthly in grams, monthly prevalence of prematurity or small for gestational age).

$time:$ child’s year of birth

$urban:$ residence area of the child (0: rural, 1: urban)

A value of coefficient for the interaction of time and residence area$(\beta_{3}$) significantly different from 0 would indicate that the trends in both groups are not parallel. This is not our case: as seen in table 2 the p values for the interactions are 0.12, 0.36 and 0.06 for birth weight, prematurity and small for gestational age, respectively for 2008 policy analysis, indicating that there is no significant deviation from parallelism in the three cases. Similar results found for the analysis of parallel trends for 2010 policy (Table S2).

The same can be visually verified in Figures S3, S4 and S5, which show the regression lines of the average birth weights, prevalence of prematurity and SGA by year of birth before and after the enactment of each law. The regression lines are parallel before the policy.

**Table S2. Regression analysis to evaluate trends**

|  |  | **Birth weight (grams)** | | **Prematurity (x 100)** | | **SGA (x 100)** | |
| --- | --- | --- | --- | --- | --- | --- | --- |
|  |  | **Coef.** | **p-value** | **Coef.** | **p-value** | **Coef.** | **p-value** |
| ***2008 policy*** | |  |  |  |  |  |  |
|  | Urban | 158.85 | 0.00 | 0.79 | 0.01 | -13.02 | 0.00 |
|  | Time | 5.59 | 0.01 | -0.23 | 0.01 | -1.19 | 0.00 |
|  | Time x urban | -4.26 | 0.12 | 0.12 | 0.36 | 0.46 | 0.06 |
|  | Constant | 3099.42 | 0.00 | 4.65 | 0.00 | 30.28 | 0.00 |
| ***2010 policy*** | |  |  |  |  |  |  |
|  | Urban | 148.85 | 0.00 | 1.36 | 0.00 | -11.90 | 0.00 |
|  | Time | 6.22 | 0.00 | 0.08 | 0.22 | -1.03 | 0.00 |
|  | Time x urban | -0.04 | 0.99 | -0.16 | 0.10 | 0.26 | 0.27 |
|  | Constant | 3108.65 | 0.00 | 3.92 | 0.00 | 27.72 | 0.00 |

S5. Evaluation of the relationship between altitude and birth outcomes

Spline variables with cubic term were created, which were related to birth weight, prematurity rates and SGA. The graphs show the relationship between outcomes and altitude, which allowed us to determine: a) for birth weight, altitude would enter the multiple model through a linear relationship (figure S6), b) for the prematurity rate, the multiple model would consider a linear and quadratic effect (figure S7) and c) for the small rate for gestational age, a linear effect would be considered in the multiple model (figure S8).

S6. Linear model of mixed effects

Next, we will present the linear model of mixed effect according to the anti-tobacco law.

**Table S3. Linear models of mixed effects for birth weight**

| **Variables** | | | **2008 policy** | |  | **Sensitivity analysis** | |
| --- | --- | --- | --- | --- | --- | --- | --- |
|  |  |  | **Coef.** | **p-value** |  | **Coef.** | **p-value** |
| Law | | | 14.06 | 0.00 |  | 14.19 | 0.00 |
| Urban | | | 5.98 | 0.27 |  | 1.02 | 0.85 |
| Urban∙Law | | | -3.10 | 0.08 |  | 0.85 | 0.63 |
| Male | | | 93.50 | 0.00 |  | 94.45 | 0.00 |
| *Place of delivery* | | |  |  |  |  |  |
|  | Hospital | | Ref |  |  | Ref |  |
|  | Centre of health | | 25.48 | 0.00 |  | 19.24 | 0.00 |
|  | Health post | | 28.17 | 0.00 |  | 12.74 | 0.00 |
|  | Consulting room | | 91.88 | 0.00 |  | 92.20 | 0.00 |
|  | Home | | 39.97 | 0.00 |  | 37.56 | 0.00 |
|  | Other | | 37.16 | 0.00 |  | 28.88 | 0.00 |
| *Person that assisted the delivery* | | |  |  |  |  |  |
|  | Health professional | | Ref |  |  | Ref |  |
|  | Health technician | | -27.11 | 0.00 |  | -26.10 | 0.00 |
|  | Midwife | | -32.29 | 0.00 |  | -27.81 | 0.00 |
|  | Another person | | -106.42 | 0.00 |  | -115.40 | 0.00 |
| *Year of birth* | |  |  |  |  |  |  |
|  | *For 2008* | *For Sensitivity* |  |  |  |  |  |
|  | 2005 | 2007 | Ref |  |  | Ref |  |
|  | 2006 | 2008 | -5.14 | 0.00 |  | -2.30 | 0.07 |
|  | 2007 | 2009 | 2.47 | 0.11 |  | 0.80 | 0.52 |
|  | 2008 | 2010 | -3.30 | 0.05 |  | -6.73 | 0.00 |
|  | 2009 | 2011 | -5.76 | 0.01 |  | -2.02 | 0.34 |
|  | 2010 | 2012 | -1.88 | 0.38 |  | 2.11 | 0.32 |
|  | 2011 | 2013 | 0.31 | 0.89 |  | 8.57 | 0.00 |
| Maternal age | | | 27.13 | 0.00 |  | 28.86 | 0.00 |
| Maternal age (square) | | | -0.45 | 0.00 |  | -0.48 | 0.00 |
| *Level of education* | | |  |  |  |  |  |
|  | No education | | Ref |  |  | Ref |  |
|  | Primary | | 52.67 | 0.00 |  | 46.34 | 0.00 |
|  | Secondary | | 83.04 | 0.00 |  | 77.63 | 0.00 |
|  | Superior non-university | | 91.94 | 0.00 |  | 87.20 | 0.00 |
|  | Higher university | | 83.26 | 0.00 |  | 77.23 | 0.00 |
| *Marital status* | |  |  |  |  |  |  |
|  | Cohabitation | | Ref |  |  | Ref |  |
|  | Married | | 4.16 | 0.00 |  | 2.39 | 0.00 |
|  | Previously joined | | -30.42 | 0.00 |  | -34.78 | 0.00 |
|  | Single | | -44.62 | 0.00 |  | -38.33 | 0.00 |
| *Number of pregnancies* | | |  |  |  |  |  |
|  | 1 |  | Ref |  |  | Ref |  |
|  | 2 |  | 57.35 | 0.00 |  | 55.46 | 0.00 |
|  | 3 |  | 75.51 | 0.00 |  | 73.31 | 0.00 |
|  | 4 |  | 92.67 | 0.00 |  | 89.32 | 0.00 |
|  | 5 |  | 107.16 | 0.00 |  | 106.49 | 0.00 |
|  | 6 |  | 118.55 | 0.00 |  | 116.27 | 0.00 |
|  | 7 |  | 124.79 | 0.00 |  | 127.75 | 0.00 |
|  | 8 |  | 140.11 | 0.00 |  | 137.56 | 0.00 |
|  | 9 |  | 145.32 | 0.00 |  | 150.35 | 0.00 |
|  | 10 |  | 163.24 | 0.00 |  | 160.27 | 0.00 |
|  | 11 |  | 169.34 | 0.00 |  | 172.09 | 0.00 |
|  | 12 |  | 185.12 | 0.00 |  | 179.33 | 0.00 |
|  | 13 |  | 180.31 | 0.00 |  | 183.49 | 0.00 |
|  | 14 |  | 269.66 | 0.00 |  | 270.19 | 0.00 |
|  | 15 and more |  | 198.52 | 0.00 |  | 197.31 | 0.00 |
| *Poverty (quintiles)* | | |  |  |  |  |  |
|  | Richest |  | Ref |  |  | Ref |  |
|  | Richer |  | -18.03 | 0.10 |  | -14.07 | 0.19 |
|  | Middle |  | -24.09 | 0.03 |  | -25.35 | 0.02 |
|  | Poorer |  | -68.54 | 0.00 |  | -65.35 | 0.00 |
|  | Poorest |  | -106.09 | 0.00 |  | -102.47 | 0.00 |
| Altitude of district of residence | | | -0.05 | 0.00 |  | -0.05 | 0.00 |
| Constant | |  | 2788.33 | 0.00 |  | 2772.87 | 0.00 |
| /sigma_u | |  | 81.77 |  |  | 79.54 |  |
| /sigma_e | |  | 458.66 |  |  | 463.59 |  |
| rho | |  | 0.03 |  |  | 0.03 |  |

S7. Logistic model of mixed effects

Next, mixed effects logistic model according to the anti-tobacco law for prematurity and small for gestational age.

**Table S4. Mixed effects Logistic model for prematurity**

| **Variables** | | | **2008 policy** | |  | **Sensitivity analysis** | |
| --- | --- | --- | --- | --- | --- | --- | --- |
|  |  |  | **Coef.** | **p-value** |  | **Coef.** | **p-value** |
| Law | |  | -0.07 | 0.00 |  | -0.05 | 0.00 |
| Urban | |  | 0.00 | 0.90 |  | 0.00 | 0.93 |
| Male | |  | 0.11 | 0.00 |  | 0.12 | 0.00 |
| *Place of delivery* | | |  |  |  |  |  |
|  | Hospital |  | Ref. |  |  | Ref. |  |
|  | Centre of health | | -1.25 | 0.00 |  | -1.29 | 0.00 |
|  | Health post |  | -0.97 | 0.00 |  | -0.94 | 0.00 |
|  | Consulting room | | -1.06 | 0.00 |  | -1.29 | 0.00 |
|  | Home |  | -0.63 | 0.00 |  | -0.63 | 0.00 |
|  | Other |  | -0.54 | 0.00 |  | -0.49 | 0.00 |
| *Person that assisted the delivery* | | |  |  |  |  |  |
|  | Health professional | | Ref. |  |  | Ref. |  |
|  | Health technician | | 0.20 | 0.00 |  | 0.26 | 0.00 |
|  | Midwife | | 0.06 | 0.05 |  | 0.11 | 0.00 |
|  | Another person | | 0.33 | 0.00 |  | 0.41 | 0.00 |
| *Year of birth* | |  |  |  |  |  |  |
|  | *For 2008* | *For Sensitivity* |  |  |  |  |  |
|  | 2005 | 2007 | Ref. |  |  | Ref. |  |
|  | 2006 | 2008 | -0.07 | 0.00 |  | 0.04 | 0.01 |
|  | 2007 | 2009 | -0.10 | 0.00 |  | -0.06 | 0.00 |
|  | 2008 | 2010 | -0.03 | 0.03 |  | -0.02 | 0.12 |
|  | 2009 | 2011 | -0.10 | 0.00 |  | -0.04 | 0.03 |
|  | 2010 | 2012 | -0.10 | 0.00 |  | 0.00 | 0.95 |
|  | 2011 | 2013 | -0.11 | 0.00 |  | -0.08 | 0.00 |
| Maternal age | |  | -0.14 | 0.00 |  | -0.15 | 0.00 |
| Maternal age (square) | | | 0.00 | 0.00 |  | 0.00 | 0.00 |
| *Level of education* | | |  |  |  |  |  |
|  | No education |  | Ref. |  |  | Ref. |  |
|  | Primary |  | -0.06 | 0.00 |  | -0.04 | 0.03 |
|  | Secondary |  | -0.08 | 0.00 |  | -0.06 | 0.00 |
|  | Superior non-university | | -0.03 | 0.11 |  | -0.03 | 0.20 |
|  | Higher university | | 0.07 | 0.00 |  | 0.10 | 0.00 |
| *Marital status* | |  |  |  |  |  |  |
|  | Cohabitation |  | Ref. |  |  | Ref. |  |
|  | Married |  | 0.03 | 0.00 |  | 0.02 | 0.01 |
|  | Previously joined | | 0.20 | 0.00 |  | 0.22 | 0.00 |
|  | Single |  | 0.32 | 0.00 |  | 0.27 | 0.00 |
| *Number of pregnancies* | | |  |  |  |  |  |
|  | 1 |  | Ref. |  |  | Ref. |  |
|  | 2 |  | 0.06 | 0.00 |  | 0.07 | 0.00 |
|  | 3 |  | 0.17 | 0.00 |  | 0.18 | 0.00 |
|  | 4 |  | 0.20 | 0.00 |  | 0.24 | 0.00 |
|  | 5 |  | 0.18 | 0.00 |  | 0.24 | 0.00 |
|  | 6 |  | 0.15 | 0.00 |  | 0.21 | 0.00 |
|  | 7 |  | 0.10 | 0.00 |  | 0.17 | 0.00 |
|  | 8 |  | 0.05 | 0.20 |  | 0.10 | 0.01 |
|  | 9 and more |  | -0.08 | 0.02 |  | -0.02 | 0.51 |
| Poverty (quintiles) | | |  |  |  |  |  |
|  | Richest |  | Ref. |  |  | Ref. |  |
|  | Richer |  | 0.11 | 0.05 |  | 0.08 | 0.12 |
|  | Middle |  | 0.10 | 0.09 |  | 0.11 | 0.05 |
|  | Poorer |  | 0.24 | 0.00 |  | 0.20 | 0.00 |
|  | Poorest |  | 0.31 | 0.00 |  | 0.31 | 0.00 |
| Altitude of district of residence | | | 0.00 | 0.02 |  | 0.00 | 0.00 |
| Altitude of district of residence (square) | | | 0.00 | 0.00 |  | 0.00 | 0.00 |
|  |  |  |  |  |  |  |  |
| Constant | |  | -1.06 | 0.00 |  | -1.10 | 0.00 |
| Risk difference marginalized over covariates | | | -0.0030 | 0.0000 |  | -0.0025 | 0.0000 |

**Table S5. Mixed effects Logistic model for small for gestational age**

| **Variables** | | | **2008 policy** | |  | **Sensitivity analysis** | |
| --- | --- | --- | --- | --- | --- | --- | --- |
|  |  |  | **Coef.** | **p-value** |  | **Coef.** | **p-value** |
| Law | |  | 0.00 | 0.55 |  | 0.00 | 0.65 |
| Urban | |  | -0.05 | 0.05 |  | -0.03 | 0.12 |
| Male | |  | 0.20 | 0.00 |  | 0.18 | 0.00 |
| *Place of delivery* | | |  |  |  |  |  |
|  | Hospital |  | Ref. |  |  | Ref. |  |
|  | Centre of health | | 0.01 | 0.02 |  | 0.03 | 0.00 |
|  | Health post |  | -0.01 | 0.23 |  | 0.04 | 0.00 |
|  | Consulting room | | -0.11 | 0.00 |  | -0.12 | 0.00 |
|  | Home |  | 0.02 | 0.08 |  | 0.03 | 0.01 |
|  | Other |  | 0.00 | 0.94 |  | -0.02 | 0.49 |
| *Delivery care* | |  |  |  |  |  |  |
|  | Health professional | | Ref. |  |  | Ref. |  |
|  | Health technician | | 0.13 | 0.00 |  | 0.13 | 0.00 |
|  | Midwife | | 0.20 | 0.00 |  | 0.18 | 0.00 |
|  | Another person | | 0.33 | 0.00 |  | 0.34 | 0.00 |
| *Year of birth* | |  |  |  |  |  |  |
|  | *For 2008* | *For Sensitivity* |  |  |  |  |  |
|  | 2005 | 2007 | Ref. |  |  | Ref. |  |
|  | 2006 | 2008 | -0.04 | 0.00 |  | -0.05 | 0.00 |
|  | 2007 | 2009 | -0.10 | 0.00 |  | -0.05 | 0.00 |
|  | 2008 | 2010 | -0.15 | 0.00 |  | -0.09 | 0.00 |
|  | 2009 | 2011 | -0.15 | 0.00 |  | -0.15 | 0.00 |
|  | 2010 | 2012 | -0.20 | 0.00 |  | -0.20 | 0.00 |
|  | 2011 | 2013 | -0.24 | 0.00 |  | -0.23 | 0.00 |
| Maternal age | |  | -0.06 | 0.00 |  | -0.06 | 0.00 |
| Maternal age (square) | | | 0.00 | 0.00 |  | 0.00 | 0.00 |
| *Level of education* | | |  |  |  |  |  |
|  | No education |  | Ref. |  |  | Ref. |  |
|  | Primary |  | -0.19 | 0.00 |  | -0.17 | 0.00 |
|  | Secondary |  | -0.35 | 0.00 |  | -0.35 | 0.00 |
|  | Superior non-university | | -0.49 | 0.00 |  | -0.49 | 0.00 |
|  | Higher university | | -0.59 | 0.00 |  | -0.59 | 0.00 |
| *Marital status* | |  |  |  |  |  |  |
|  | Cohabitation |  | Ref. |  |  | Ref. |  |
|  | Married |  | -0.06 | 0.00 |  | -0.06 | 0.00 |
|  | Previously joined | | 0.07 | 0.01 |  | 0.10 | 0.00 |
|  | Single |  | 0.11 | 0.00 |  | 0.09 | 0.00 |
| *Number of pregnancies* | | |  |  |  |  |  |
|  | 1 |  | Ref. |  |  | Ref. |  |
|  | 2 |  | -0.35 | 0.00 |  | -0.35 | 0.00 |
|  | 3 |  | -0.44 | 0.00 |  | -0.45 | 0.00 |
|  | 4 |  | -0.48 | 0.00 |  | -0.49 | 0.00 |
|  | 5 |  | -0.50 | 0.00 |  | -0.52 | 0.00 |
|  | 6 |  | -0.53 | 0.00 |  | -0.53 | 0.00 |
|  | 7 |  | -0.51 | 0.00 |  | -0.53 | 0.00 |
|  | 8 |  | -0.58 | 0.00 |  | -0.58 | 0.00 |
|  | 9 |  | -0.54 | 0.00 |  | -0.57 | 0.00 |
|  | 10 |  | -0.61 | 0.00 |  | -0.60 | 0.00 |
|  | 11 |  | -0.63 | 0.00 |  | -0.65 | 0.00 |
|  | 12 |  | -0.59 | 0.00 |  | -0.62 | 0.00 |
|  | 13 |  | -0.72 | 0.00 |  | -0.79 | 0.00 |
|  | 14 |  | -0.92 | 0.00 |  | -0.68 | 0.00 |
|  | 15 and more |  | -0.79 | 0.00 |  | -0.93 | 0.00 |
| *Poverty (quintiles)* | | |  |  |  |  |  |
|  | Richest |  | Ref. |  |  | Ref. |  |
|  | Richer |  | 0.07 | 0.16 |  | 0.05 | 0.23 |
|  | Middle |  | 0.12 | 0.01 |  | 0.12 | 0.01 |
|  | Poorer |  | 0.30 | 0.00 |  | 0.29 | 0.00 |
|  | Poorest |  | 0.44 | 0.00 |  | 0.42 | 0.00 |
| Altitude of district of residence | | | 0.00 | 0.00 |  | 0.00 | 0.00 |
| Constant | |  | -0.66 | 0.00 |  | -0.68 | 0.00 |
| Risk difference marginalized over covariates | | | -0.0006 | 0.5460 |  | -0.0004 | 0.6490 |

**S7. Others sensitivity analysis**

In addition to the sensitivity analysis that evaluated the effect of the law in 2010, we conducted three more evaluations:

1. Excluding child variables (place of delivery, delivery care)
2. Using lag time (6 months later, 12 months later)

The table S6 show the three mixed effects models.

**Table S6. Mixed effects models: Differences and 95% CI**

| **Outcome** | **No child variables** | **6 months later** | **12 months later** |
| --- | --- | --- | --- |
| Birth weight (g) | -4.29 (-7.75 , -0.83) | -2.79 (-6.26 , 0.68) | 0.47 (-2.98 , 3.91) |
| Prematurity (%) | -0.28 (-0.39 , -0.17) | -0.44 (-0.60 , -0.28) | -0.13 (-0.24 , -0.02) |
| Small for gestational age (%) | 0.03 (-0.16 , 0.23) | 0.48 (0.25 , 0.72) | -0.23 (-0.41 , -0.05) |

Models adjusted for the following variables: mother’s age, level of education, marital status, and parity, newborn year of birth, gender, area of residence, poverty quintiles, and altitude.

**Legends for supplementary figures**

**Figure S1: Trend prevalence of smoking throughout life, in the last year and in the last 30 days in urban areas of Peru.**

**Figure S4. Average weight trends at birth before the anti-tobacco policy.**

**Figure S5. Prevalence prematurity trends at birth before the anti-tobacco policy.**

**Figure S6. Prevalence small for gestational age trends at birth before the anti-tobacco policy.**

**Figure S7. Relationship between birth weight and altitude of the district of residence.**

**Figure S8. Relationship between prematurity proportion and altitude of the district of residence.**

**Figure S9. Relationship between small for gestational age proportion and altitude of residence district.**

1. Decreto Supremo N° 015-2008 SA. Reglamento de la ley n^o^ 28705, Ley General para la Prevención y Control de los riesgos del consumo del tabaco [↑](#footnote-ref-1)
2. Decreto Supremo No. 001-2010-SA. Se modifican artículos del Reglamento de la Ley No. 28705 [↑](#footnote-ref-2)
3. Decreto Supremo No. 001-2011-SA. Modifican el Reglamento de la Ley N^o^ 28705 [↑](#footnote-ref-3)
4. Resolución Ministerial No. 415-2012/MINSA. Aprueban Norma Técnica de Salud para Inspecciones de ambientes 100% libres de humo de tabaco [↑](#footnote-ref-4)
