# Supplementary figures and images for: The impact of anti-tobacco legislation on birth weight in Peru
Source: Glob Health Res Policy. 2020 Feb 28;5:5. doi: 10.1186/s41256-020-00136-5 (PMC7048150; doi:10.1186/s41256-020-00136-5)

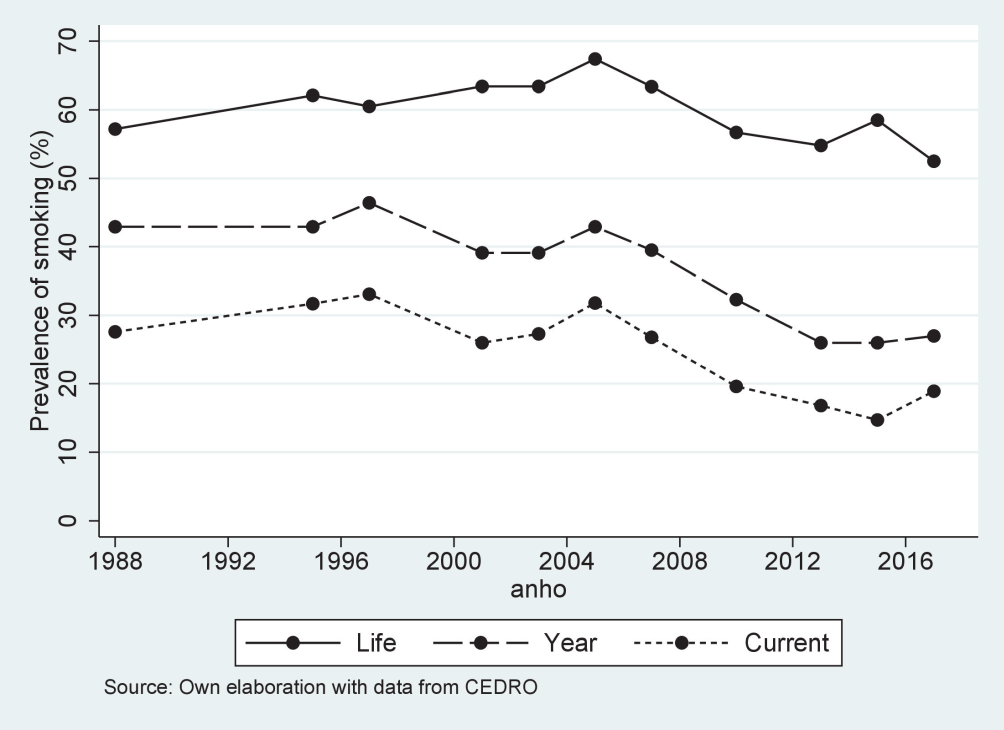

Supplement: Supplementary file 1 — Additional file 1. Supporting Information. [file 41256_2020_136_MOESM1_ESM.zip › Figure S1-sup.tif]

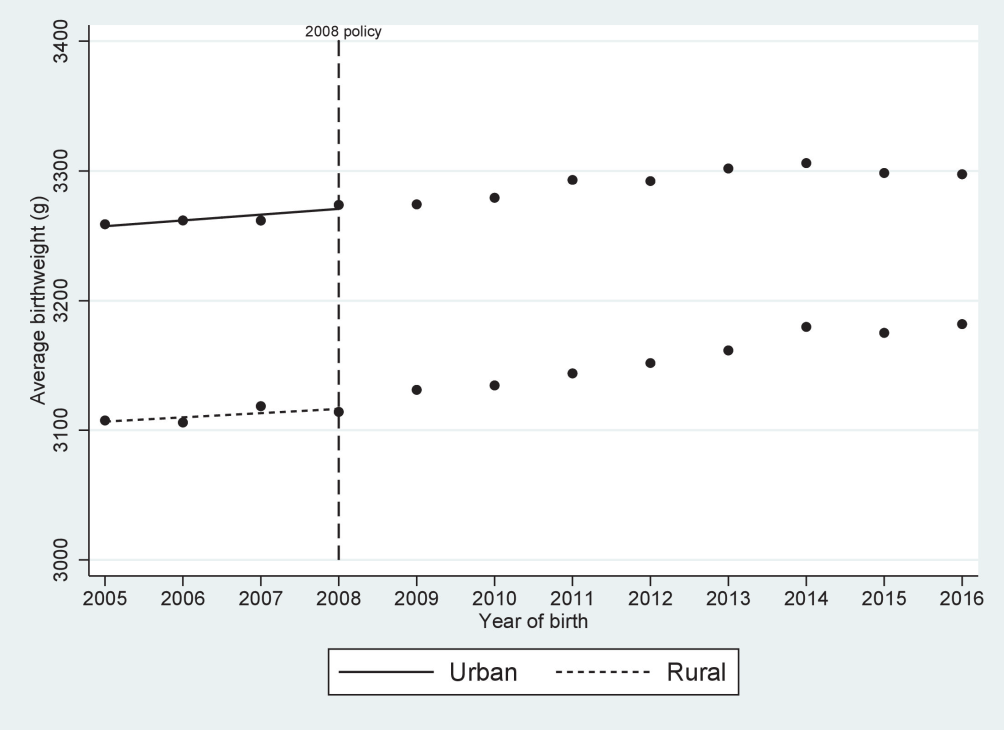

Supplement: Supplementary file 1 — Additional file 1. Supporting Information. [file 41256_2020_136_MOESM1_ESM.zip › Figure S4-sup.tif]

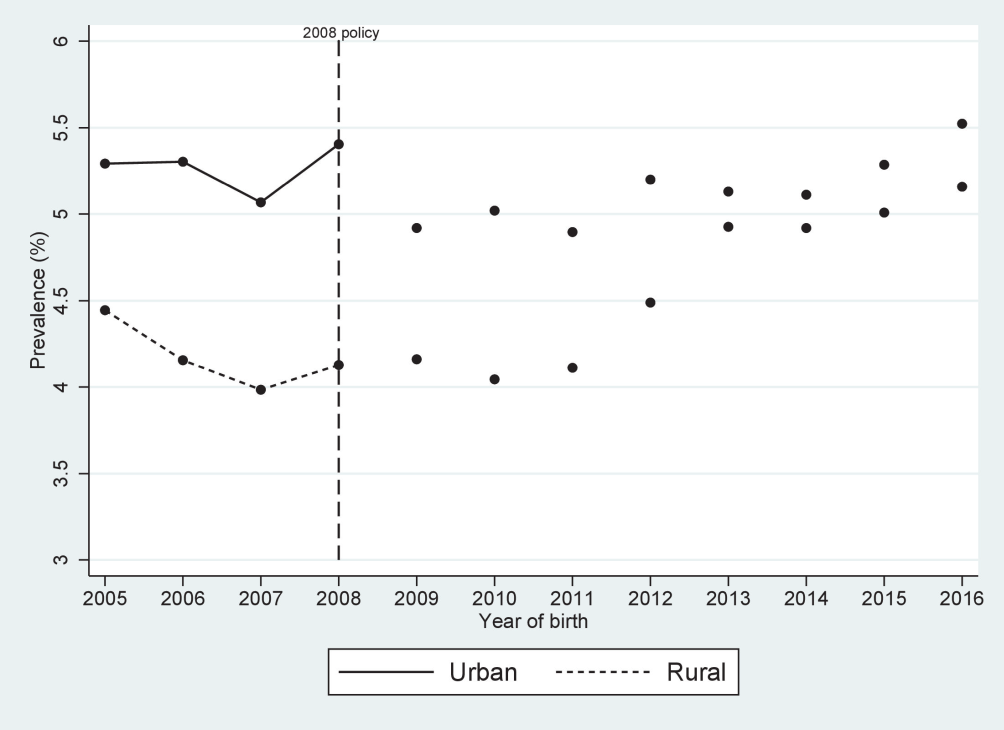

Supplement: Supplementary file 1 — Additional file 1. Supporting Information. [file 41256_2020_136_MOESM1_ESM.zip › Figure S5-sup.tif]

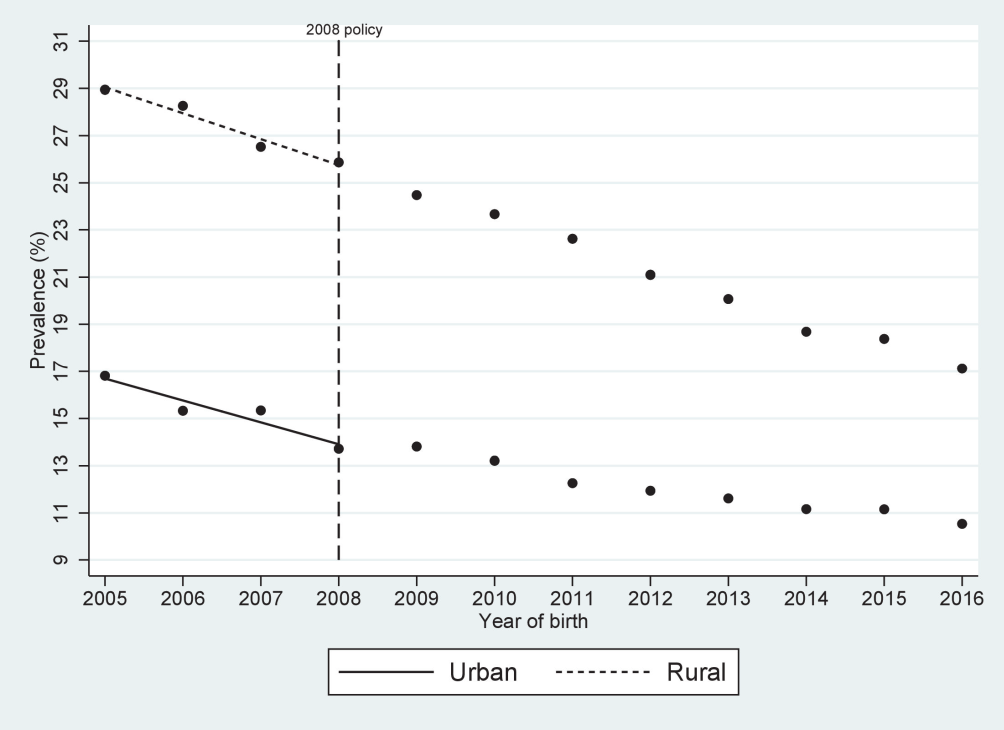

Supplement: Supplementary file 1 — Additional file 1. Supporting Information. [file 41256_2020_136_MOESM1_ESM.zip › Figure S6-sup.tif]

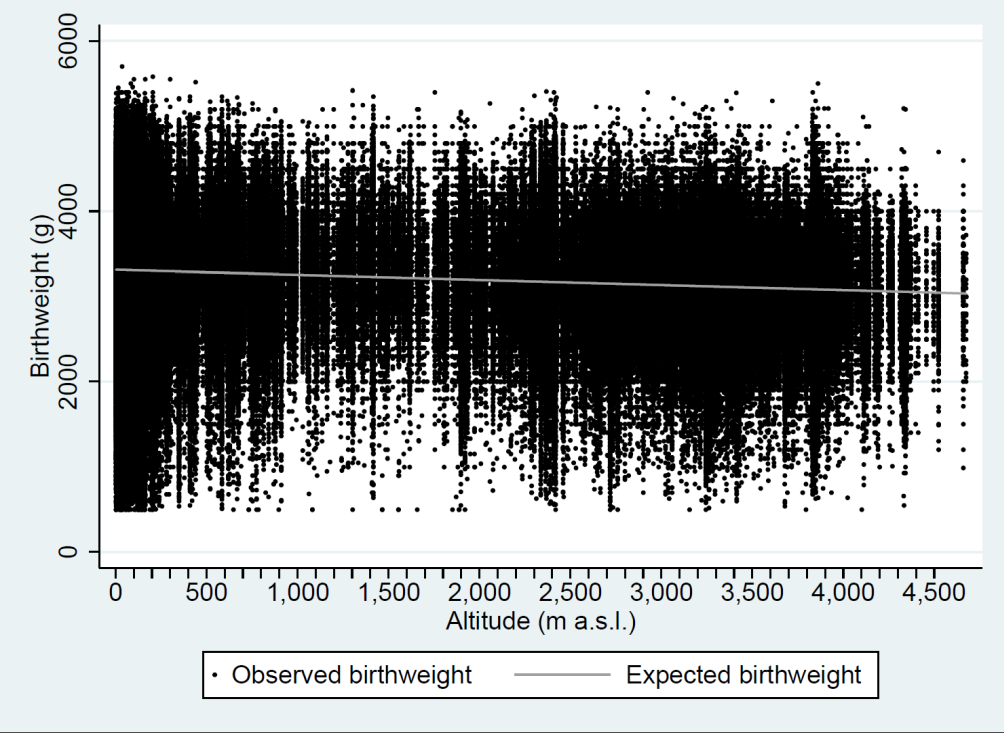

Supplement: Supplementary file 1 — Additional file 1. Supporting Information. [file 41256_2020_136_MOESM1_ESM.zip › Figure S7-sup.tif]

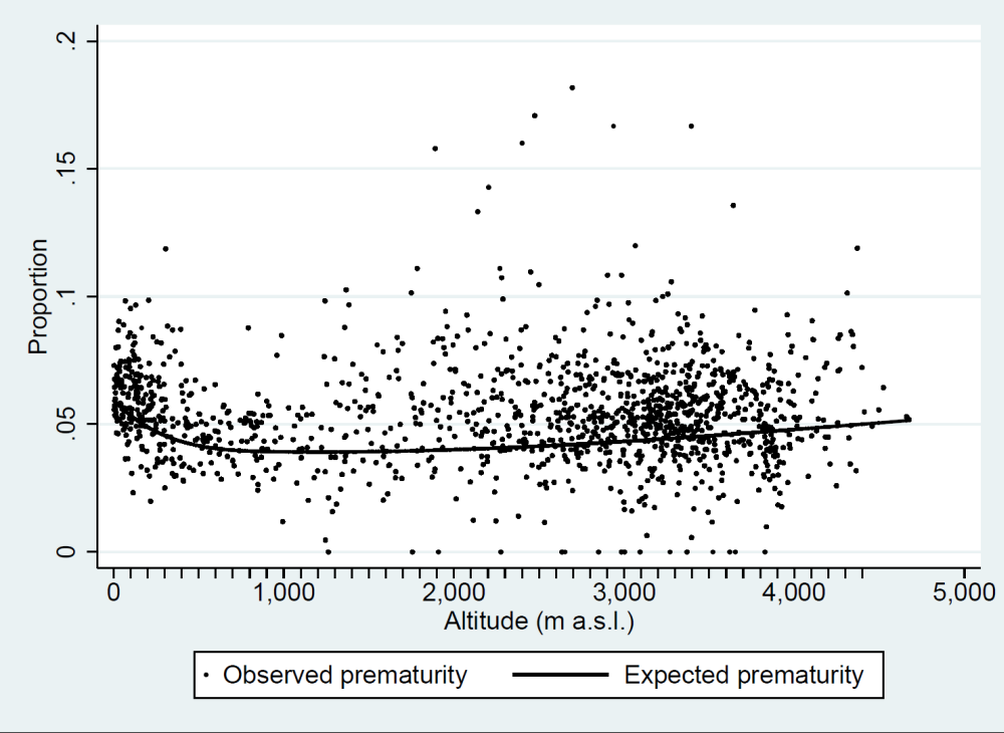

Supplement: Supplementary file 1 — Additional file 1. Supporting Information. [file 41256_2020_136_MOESM1_ESM.zip › Figure S8-sup.tif]

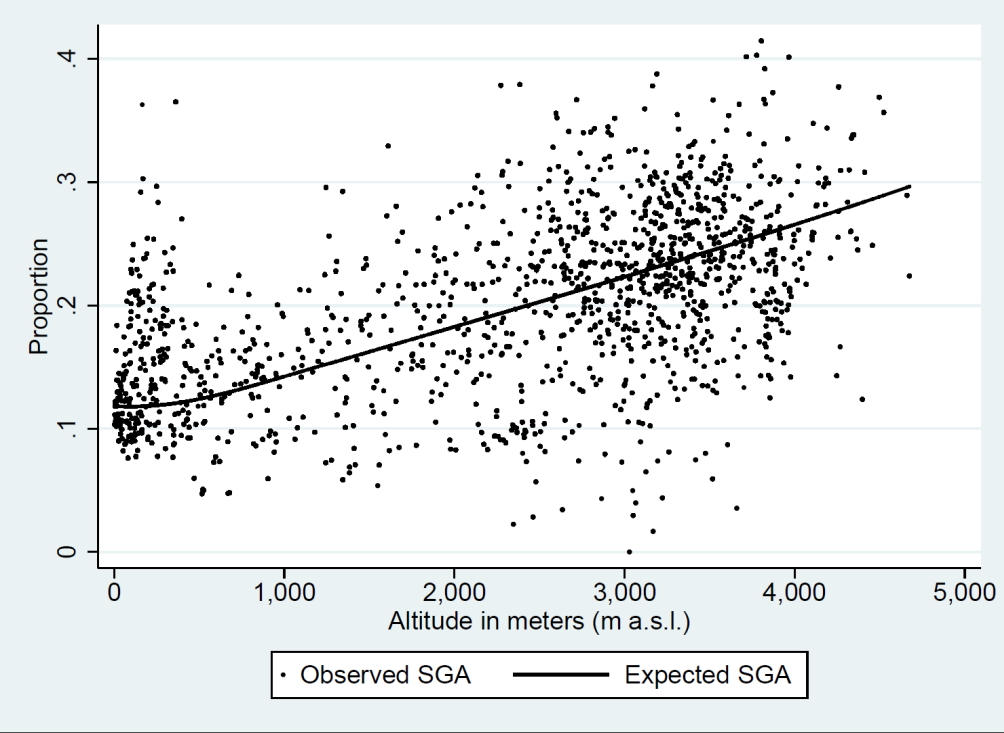

Supplement: Supplementary file 1 — Additional file 1. Supporting Information. [file 41256_2020_136_MOESM1_ESM.zip › Figure S9-sup.tif]
